# Ezrin Phosphorylation at T567 Modulates Cell Migration, Mechanical Properties, and Cytoskeletal Organization

**DOI:** 10.3390/ijms21020435

**Published:** 2020-01-09

**Authors:** Xiaoli Zhang, Luis R. Flores, Michael C. Keeling, Kristina Sliogeryte, Núria Gavara

**Affiliations:** School of Engineering and Material Science, Queen Mary University of London, Mile End Road, London E1 4NS, UK; xiaoli.zhang@qmul.ac.uk (X.Z.); l.r.pereiradocarmoflores@qmul.ac.uk (L.R.F.); m.c.keeling@qmul.ac.uk (M.C.K.); k.sliogeryte@qmul.ac.uk (K.S.)

**Keywords:** ezrin, cell mechanics, cell migration, image processing and quantification, actin

## Abstract

Ezrin, a member of the ERM (ezrin/radixin/moesin) family of proteins, serves as a crosslinker between the plasma membrane and the actin cytoskeleton. By doing so, it provides structural links to strengthen the connection between the cell cortex and the plasma membrane, acting also as a signal transducer in multiple pathways during migration, proliferation, and endocytosis. In this study, we investigated the role of ezrin phosphorylation and its intracellular localization on cell motility, cytoskeleton organization, and cell stiffness, using fluorescence live-cell imaging, image quantification, and atomic force microscopy (AFM). Our results show that cells expressing constitutively active ezrin T567D (phosphomimetic) migrate faster and in a more directional manner, especially when ezrin accumulates at the cell rear. Similarly, image quantification results reveal that transfection with ezrin T567D alters the cell’s gross morphology and decreases cortical stiffness. In contrast, constitutively inactive ezrin T567A accumulates around the nucleus, and although it does not impair cell migration, it leads to a significant buildup of actin fibers, a decrease in nuclear volume, and an increase in cytoskeletal stiffness. Finally, cell transfection with the dominant negative ezrin FERM domain induces significant morphological and nuclear changes and affects actin, microtubules, and the intermediate filament vimentin, resulting in cytoskeletal fibers that are longer, thicker, and more aligned. Collectively, our results suggest that ezrin’s phosphorylation state and its intracellular localization plays a pivotal role in cell migration, modulating also biophysical properties, such as membrane–cortex linkage, cytoskeletal and nuclear organization, and the mechanical properties of cells.

## 1. Introduction

Ezrin, a member of the ERM (ezrin, radixin, moesin) family, serves as a cross-linker between the plasma membrane and the actin cytoskeleton [1]. Through its interaction with transmembrane proteins and the underlying cytoskeleton, it helps provide structural support to strengthen the cell cortex. A plethora of molecular biology studies have focused on characterizing the monomeric or oligomeric configuration of ezrin when bound to the cellular membrane or its binding rates and unbinding forces to the membrane and/or the actin cortex, especially in terms of ezrin’s activation state [2,3,4,5,6,7]. Ezrin is also important in the biological regulation of signaling pathways in the vicinity of the cellular membrane, allowing for optimal signaling from membrane-associated receptors, such as hepatocyte and epidermal growth factor receptors, to their respective downstream effector pathways, e.g., Cdc42, MAP [8]. Of note, ezrin signaling is believed to extend beyond the cortical cell region, and includes regulatory roles in RhoA-mediated contractility, maturation of focal adhesions, or Ras-induced transformation of cells [9]. Given the broad extent of its regulatory function as well as its connection to the cell’s cytoskeleton, it can be hypothesized that ezrin may play a wider role than that of a membrane linker and thus also influence the cellular state and function.

A body of work has focused on the role of ezrin in cell morphology and function, especially on highly polarized cells, such as those found in epithelial monolayers [6,7,10,11,12]. In this connection, there is a broad consensus in that ezrin activation induces its redistribution within the cell, which then leads to gross cellular morphological changes. For example, ezrin’s activation via HGF-receptor stimulation leads to the disappearance of microvilli, changes in the cytoskeleton, and increased cell migration [11]. Similarly, ezrin activation via increases in the PIP2 concentration leads to the recruitment of additional ezrin to the apical cortex and increases in membrane tension [7]. On the contrary, loss of ezrin activation leads to gross changes in cell morphology and behavior, with a reduction in the cell spread area, scattering, migratory ability, and tubulogensis [7,10,11,12]. Together, these results suggest that changes in ezrin’s activation state or its intracellular localization influence cell behavior and especially its migratory disposition.

In this connection, a number of clinical studies have highlighted that ezrin is involved in tumor progression, especially metastasis [13], in osteosarcoma [14], lung cancer [15], tongue squamous cell carcinoma [16], breast cancer [17], pancreatic cancer [18], and uterine cervical cancer [19]. The level of ezrin expression and its activation state, as well as the subcellular localization of ezrin, are important factors in tumor progression. Several clinical studies have linked high expression of ezrin or phosphorylation of ezrin at threonine T567 with poor outcomes in patients suffering from a wide variety of cancers. Furthermore, it is reported that T567 hyperphosphorylation of ezrin is tightly correlated to an invasive phenotype of clinical hepatocellular carcinomas [20]. Similarly, phosphorylated ezrin was found at the invasive front of large metastatic lesions in osteosarcoma [21]. These findings have thus pointed towards the targeting of ezrin (or its activation) as a means to reduce cancer cell migration and tumor metastasis. Accordingly, a recent promising work on a mouse model of breast cancer has shown that systemic treatment with an ezrin inhibitor reduces the migration of cancer cells, also diminishing the metastatic burden to the axillary lymph nodes and the lungs [22].

Despite the body of work on ezrin at the molecular, cellular, and biomedical levels, little is still known on the extent to which ezrin activation and its intracellular localization lead cells to adopt biophysical characteristics that are permissive of cell migration and thus typically linked to cancer metastasis. The switch towards a motile phenotype is not only associated with increased intracellular polarization (along the migratory axis) of specific regulatory proteins and kinases [23], but it may also be accompanied by changes in the organization of the cytoskeleton and the cell’s mechanical state [24]. Obtaining this kind of information will allow an extension of the established regulatory role of ezrin in the membrane vicinity towards a broader modulation of the whole-cell morphology and biophysical phenotypes that may then enable metastasis and cancer progression.

In this study, we used a combination of biophysical tools to understand how different activation states of ezrin at T567 modulate cell migration, leading to distinct cytoskeleton organization and mechanical properties. We showed that when ezrin is constitutively active and preferentially located at the cell rear, cells migrate faster and in a more directional manner. Simultaneously, cells displayed altered cellular and nuclear morphology, and a softer actin cortex. On the other hand, cells in which ezrin’s role as a membrane–actin linker was impaired displayed altered cytoskeletal organization and mechanical properties, even though migration was not impaired. Collectively, our results suggest that ezrin’s phosphorylation state plays a pivotal role in cells’ biophysical behaviors that are typically associated with metastasis and cancer progression.

## 2. Results

### 2.1. The Effect of Ezrin’s Phosphorylation State on Cell Migration and Stiffness

We chose to study the biological and biomechanical function of ezrin in NIH3T3 fibroblast cells, which were derived from Swiss albino mouse embryo tissue. To analyze the role of ezrin’s phosphorylation state, we transfected cells with plasmids containing different mutations of ezrin: (1) Wild-type ezrin, which can switch between active and inactive modes; (2) the phosphomimetic mutant ezrin T567D, which acts as a constitutively active ezrin; and (3) the phosphodeficient mutant ezrin T567A, which is constitutively inactive. Apart from those, we also transfected cells with truncated ezrin at the FERM domain, which only contains the plasma membrane-binding domain and is thought to act as a competitive inhibitor for all endogenous ERM proteins. The exogenous proteins were tagged with RFP (FERM mutation plasmid) or EGFP (the remaining plasmids). A previous study using a similar transfection strategy but a different cell line stated that the expression levels of exogenous ezrin are at least an order of magnitude larger than those of endogenous ezrin, thus likely ruling out the fact that the endogenous protein may have a strong influence on the observed behavior after transfection [25]. We verified that in our transfection experiments, similar conditions were achieved. In particular, we found that the amount of exogenous ezrin relative to endogenous ezrin was estimated to be up to 20-fold from the quantification of immunostaining images comparing cells expressing ezrin-EGFP and cells without transfection, all of which were fixed and stained with ezrin antibody (Figures S2 and S3).

First, we investigated the potential role of ezrin’s phosphorylation state on cell migration by performing long-term live cell imaging on transfected cells. Time lapses of fluorescent images for individual cells were taken every 5 min over a period of 12 h. Throughout this study, and following an approach previously used by others, we considered cells transfected with wild-type ezrin plasmid as controls for our experiments [26]. As verification, we transfected cells with EGFP-plasmid only and showed that neither ezrin-GFP nor EGFP altered NIH3T3 cells’ motility (Figure S4).

**Figure 1:**
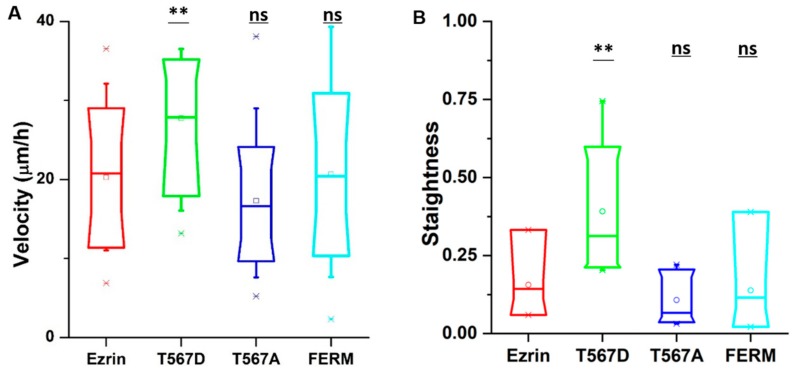
The effect of ezrin mutations on cell migration. NIH 3T3 cells were transfected with wild-type ezrin (red), ezrin T567D (green), ezrin T567A (blue), or FERM domain (cyan). Transfected cells were used for a migration experiment and (Atomic Force Microscope) AFM experiment. Box plots show the results of migration velocity (**A**) and migration directionality (**B**). Box plots extend from the 10th to the 90th percentile, whiskers from the 5th to the 95th. A total of n = 21 (ezrin), n = 45 (ezrin T567D), n = 2 (ezrin T567A), and n = 60 (FERM) cells were analyzed from n = 4 repeats. Asterisks indicate a statistical difference (* *p* < 0.05, ** *p* < 0.01, *** *p* < 0.001, obtained using Dunnett’s test against wild-type ezrin).

In our time-lapse experiments, we observed that cells transfected with active ezrin T567D had the largest migration speed (30 µm per hour) compared with cells transfected with other plasmids (Figure 1A). In addition, cells migrated in a more directional manner, following straight tracks that displayed reduced randomness (Figure 1B). In contrast, the expression of inactive ezrin T567A and dominant negative FERM domain had no effect on cell migration as compared to wild-type ezrin plasmid (Figure 1A). We also tried to assess at the single-cell level whether the effect on cell migration was dependent on the individual levels of exogenous protein. While some weak trends were observed (a positive trend for T567D and a negative trend for FERM), the very large variability in the measured cell speeds prevented us from identifying statistically significant trends (Figure S5). Because of that observation, we decided to pool all our data for each plasmid used, disregarding the individual level of exogenous protein measured for each probed cell.

### 2.2. Subcellular Distribution of Ezrin Mutants

**Figure 2:**
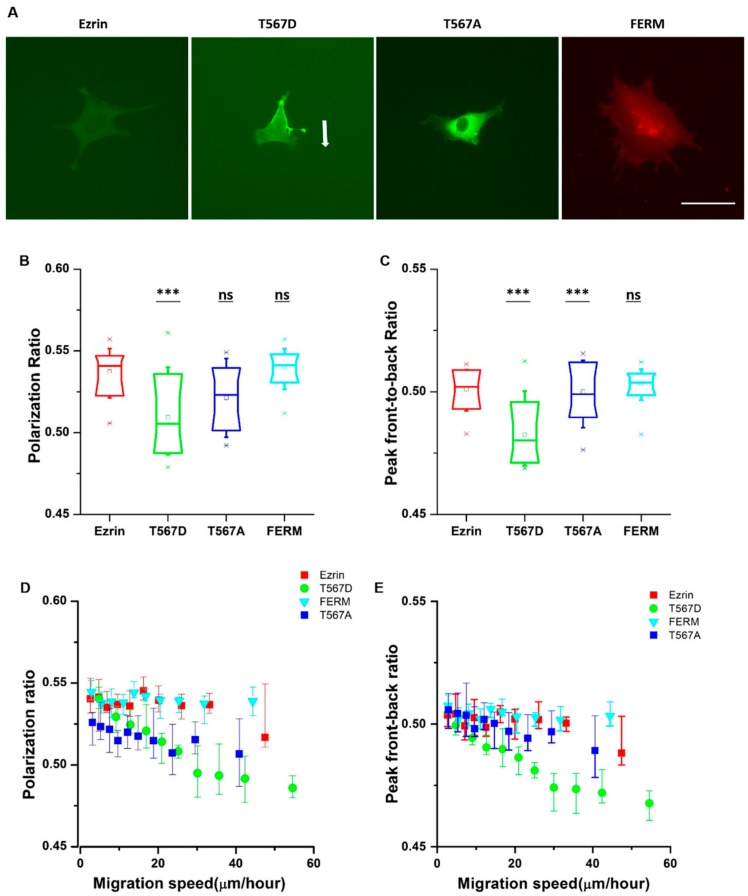
The subcellular distribution of ezrin and its mutations during migration. (**A**) Example fluorescent images of transfected cells obtained from the time-lapse videos. The example cell for ezrin T567D showed clear persistent directional migration, indicated by the arrow. The other example cells showed no clear directional migration. Scale bar 50 µm. Box plots show the results of the polarization ratio (**B**) and peak front-to-back ratio (**C**). Box plots extend from the 10th to the 90th percentile, whiskers from the 5th to the 95th. The plot shows the relationship between the cell migration velocity and the polarization ratio (**D**) and peak front-to-back ratio (**E**), error bars indicate SD. A total of n = 21 (ezrin), n = 45 (ezrin T567D), n = 52 (ezrin T567A), and n = 60 (FERM) cells were analyzed from n = 4 repeats. Asterisks indicate a statistical difference (*** *p* < 0.001, obtained using Dunnett’s test against wild-type ezrin).

From the time-lapse videos, we observed that ezrin’s intracellular distribution patterns during cell migration were different for different mutants (Figure 2A). Thus, we aimed to identify the relationship between ezrin’s intracellular distribution and the previously observed biophysical properties. Accordingly, we defined the polarization ratio and peak front-to-back ratio separately to describe the intracellular distribution during migration. The polarization ratio describes the spread of the fluorescence intensity within the cell area, with 1 meaning a full homogeneous spread and 0 concentrated at one point. The peak front-to-back ratio identifies the averaged intracellular location where most protein is found with respect to the direction of cell movement, with 1 representing the cell front and 0 the cell rear. Active ezrin T567D was the most highly localized mutant with the lowest polarization ratio of 0.51 (Figure 2B). Furthermore, its localization was preferentially at the cell rear, displaying the smallest value measured from all mutations for the peak front-to-back ratio (Figure 2C). Conversely, inactive ezrin T567A formed a well-localized ring around the nucleus (Figure 2B). Wild-type ezrin and dominant negative FERM domain displayed the broadest distribution through the cell cytoplasm, yielding the highest values for the polarization ratio of 0.54 (Figure 2B).

Since cell migration is a dynamic process, the values of the cell migration speed, polarization ratio, and peak front-to-back ratio for each individual cell change during the course of a time-lapse experiment. Therefore, we assessed whether there was a relationship between the instantaneous cell migration speed and intracellular protein distribution patterns. To do so, we pooled together the results from all frames in all videos, and plotted the instantaneous migration speed against the instantaneous protein distribution parameters. We found that there was a strong linear relationship between the migration speed and polarization ratio and peak front-to-back ratio for active ezrin T567D, that is, when active ezrin T567D accumulated at the cell rear, cells migrated faster (Figure 2D,E). Together, these results suggest that active ezrin T567D enhances cell migration by preferentially localizing at the cell rear while the presence of ezrin (in any phosphorylation state) in the vicinity of the nucleus tends to hinder cell migration.

### 2.3. The Effect of Ezrin Mutations on Cell Morphology, the Nucleus, and the Actin Cytoskeleton

**Figure 3:**
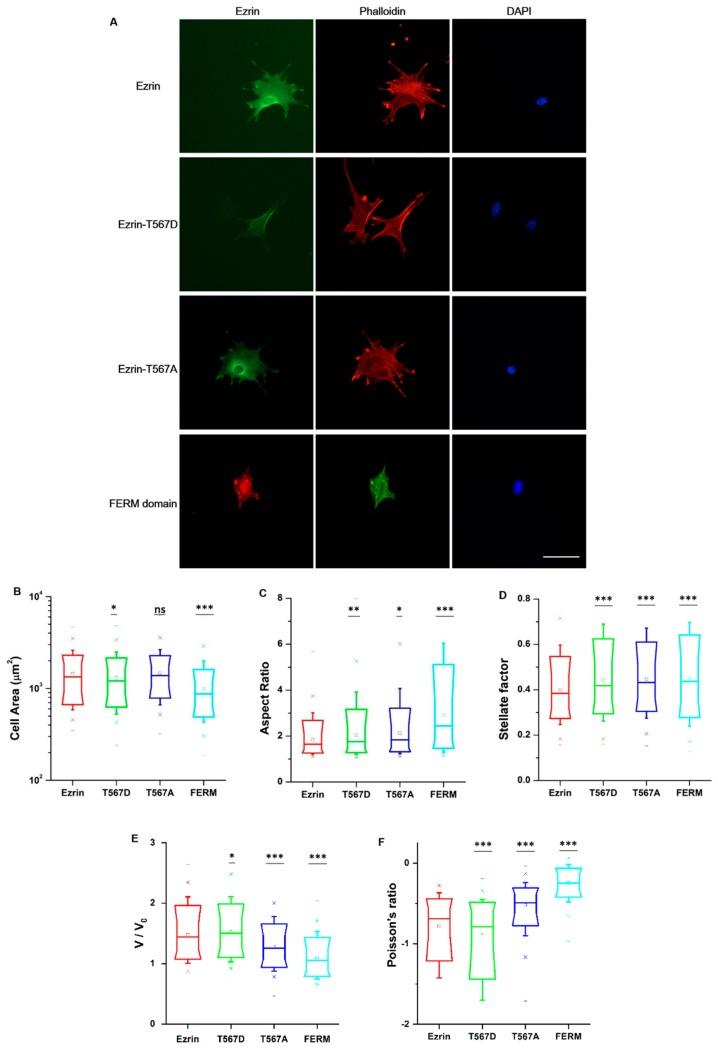
The effect of ezrin mutations on cellular and nuclear morphology. (**A**) Fluorescent images of transfected cells subsequently stained with phalloidin and 4′,6-diamidino-2-phenylindole (DAPI). Left: transfected cells expressing ezrin mutant coupled with Green Fluorescent protein (GFP) or Red Fluorescent protein (RFP); middle: actin filament stained with phalloidin; right: cell nucleus stained with DAPI. Scale bar 50 µm. Box plots show the results of the cell area (**B**) and aspect ratio (**C**), stellate factor (**D**), nuclear volume (**E**), and nuclear Poisson’s ratio (**F**). Box plots extend from the 10th to the 90th percentile, whiskers from the 5th to the 95th. A total of n = 420 (ezrin), n = 496 (ezrin T567D), n = 1235 (ezrin T567A), and n = 562 (FERM) cells were analyzed from n = 6 repeats. Asterisks indicate a statistical difference (* *p* < 0.05, ** *p* < 0.01, *** *p* < 0.001, as obtained using Dunnett’s test against wild-type ezrin).

Ezrin signaling is believed to extend beyond the cortical cell region, including regulatory roles in RhoA-mediated contractility and the maturation of focal adhesions [9]. Accordingly, we chose to assess whether ezrin’s phosphorylation state would give rise to dissimilar cell morphologies and organization of the actin cytoskeleton or the nucleus. We cultured cells at low density in unrestricted spreading conditions and then immunostained the transfected cells with phalloidin and 4′,6-diamidino-2-phenylindole (DAPI) (Figure 3A). Fluorescent images of the channels for ezrin plasmids, the actin cytoskeleton, and cell nucleus were taken using an epifluorescence microscope. First, we verified that ezrin’s intracellular distribution was in line with those previously measured in our time-lapse images. Briefly, ezrin T567D was indeed preferentially localized to the cell membrane colocalizing with actin filaments. Conversely, the FERM domain was distributed throughout the cytoplasm and especially colocalizing with the nucleus, whereas wild-type ezrin and inactive ezrin T567A were localized in the cytoplasm, with ezrin T567A preferentially concentrated as a ring around the nucleus (Figure 3A).

**Figure 4:**
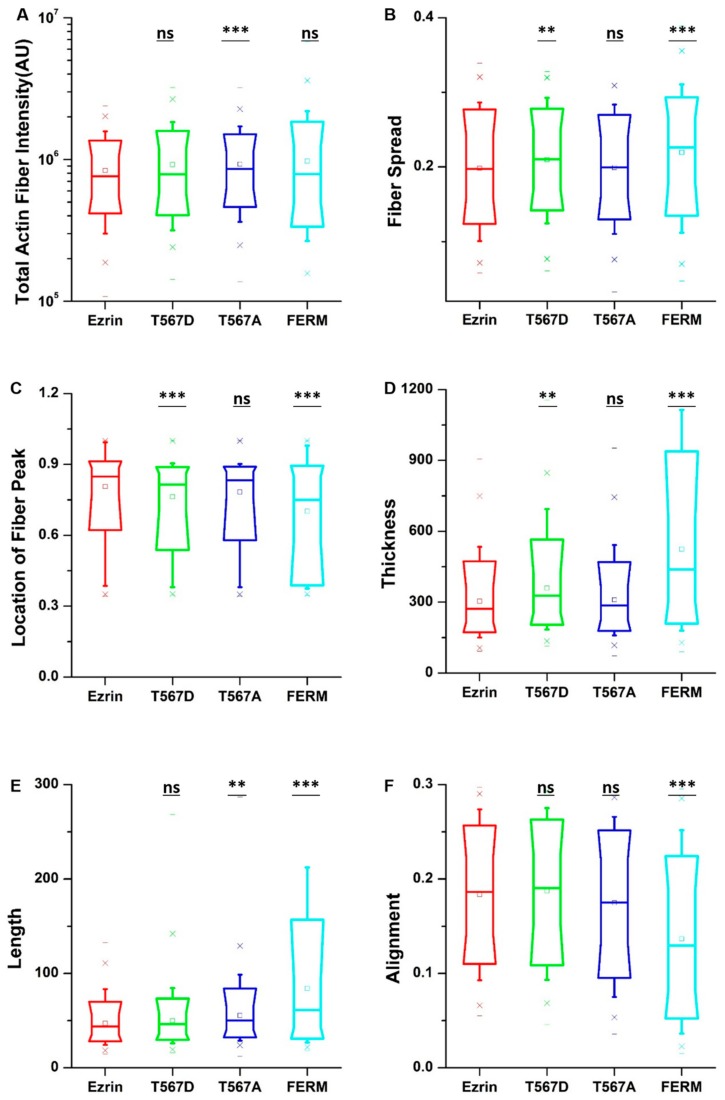
The effect of ezrin mutations on the actin cytoskeleton. Box plots show the results of the total actin fiber intensity (**A**), fiber spread (**B**), location of the fiber peak (**C**), fiber thickness (**D**), fiber length (**E**), and fiber alignment (**F**). A total of n = 420 (ezrin), n = 496 (ezrin T567D), n = 1235 (ezrin T567A), and n = 562 (FERM) cells were analyzed from n = 6 repeats. Box plots extend from the 10th to the 90th percentile, whiskers from the 5th to the 95th. Asterisks indicate a statistical difference (** *p* < 0.01, *** *p* < 0.001, obtained using Dunnett’s test against wild-type ezrin).

To obtain quantitative data on the cellular morphology, actin organization, and nuclear state, we used images obtained through the phalloidin and DAPI fluorescence channels and we input them in our image quantification pipeline [27]. Cells transfected with active ezrin T567D and FERM domain had smaller cell spread areas (Figure 3B) and larger aspect ratios (Figure 3C). Of note, transfection with T567D, T567A, and FERM all resulted in larger values for stellate factor, which reflects the tendency of cells to extend protrusions, such as blebs, lamellipodia, or filopodia (Figure 3D). Similar results have been reported by others [28,29] and likely reflect a local (for T567D) or global (for T567A and FERM) weakening of the cortex–membrane connection. We also quantified the morpho-mechanical parameters of the nucleus that help understand the mechanical connection between the nucleus and the cytoskeleton. The nuclear volume was significantly increased for T567D transfection, which reflects a stronger transmission of intracellular tension to the nucleus (Figure 3E). On the contrary, T567A and FERM transfections lead to a significant reduction in nuclear volume, rather reflecting a weaker force transmission between the cytoskeleton and the nucleus. When assessing the Poisson’s ratio of the nucleus, we verified that in all conditions, the nuclei displayed auxetic properties [30], which were significantly enhanced for T567D transfection and significantly reduced for T567A and FERM (Figure 3F). When assessing actin organization, we found that transfection with active ezrin T567D induced thicker (Figure 4D) and more spread actin fibers (Figure 4B). Conversely, transfection with inactive ezrin T567A lead to increased overall amounts of F-actin (Figure 4A). Finally, cells expressing the FERM domain displayed actin fibers that were increasingly located near the cell nucleus (Figure 4C). Furthermore, the actin cytoskeleton was organized into longer (Figure 4E), thicker (Figure 4D), and more aligned (smaller values in Figure 4F) fibers. Together, our data evidence that the phosphorylation state of ezrin leads to distinct cellular and nuclear morphologies and dissimilar actin organization.

### 2.4. The Effect of Ezrin Mutations on Tubulin and Vimentin Organization

**Figure 5:**
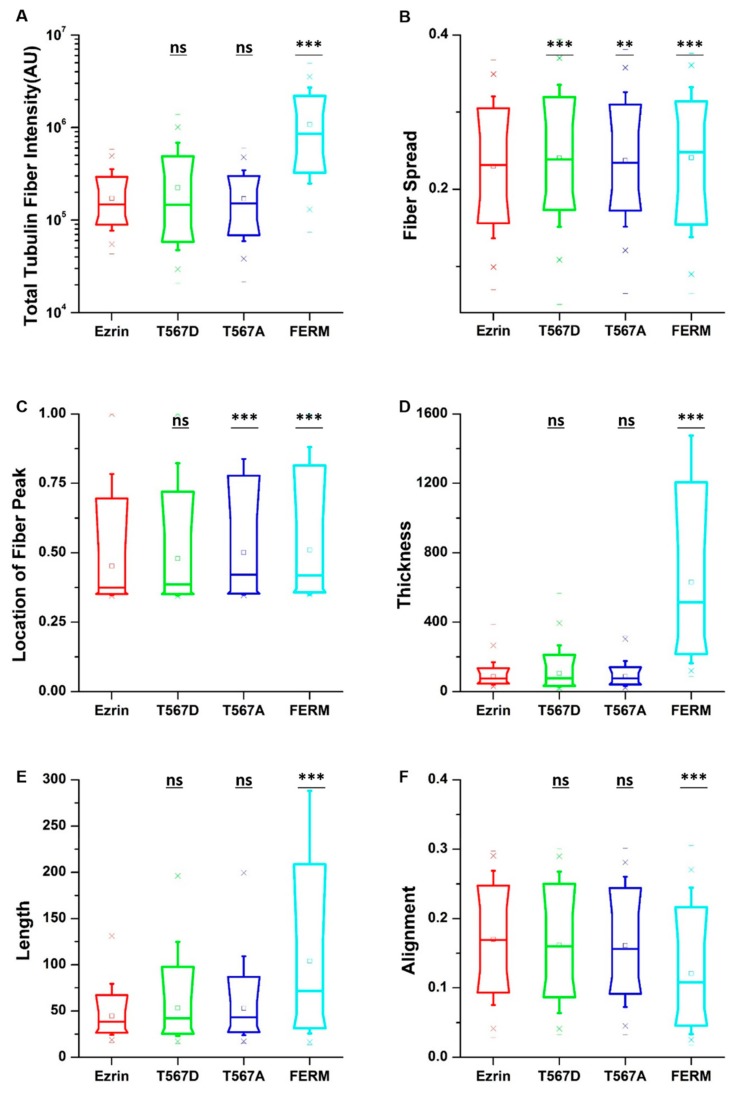
The effect of ezrin mutations on the tubulin cytoskeleton. Box plots show the results of the total tubulin fiber intensity (**A**), fiber spread (**B**), location of the fiber peak (**C**), fiber thickness (**D**), fiber length (**E**), and fiber alignment (**F**). Box plots extend from the 10th to the 90th percentile, whiskers from the 5th to the 95th. A total of n = 559 (ezrin), n = 578 (ezrin T567D), n = 526 (ezrin T567A), and n = 408 (FERM) cells were analyzed from n = 6 repeats. Asterisks indicate a statistical difference (** *p* < 0.01, *** *p* < 0.001, Dunnett’s test against wild-type ezrin).

All the biopolymers composing the cytoskeleton, i.e., actin filaments, microtubules, and intermediate filaments, work synergistically to support normal cell function. Therefore, we hypothesized that changes in the assembly of one particular cytoskeletal network would appear alongside changes in the assembly of all other networks. Having estimated the influence of ezrin mutations on the cell morphology and actin fibers, we moved to investigate its role on the tubulin and vimentin networks by replacing phalloidin staining with antibodies against tubulin and vimentin. Regardless of the plasmid transfected, microtubules were observed as a fibrous network starting from the perinuclear region and spreading randomly through the cytoplasm of the cell (Figure S6). Surprisingly, quantification of the fiber parameters revealed similar effects to those previously observed on actin fibers. Cells transfected with active ezrin T567D showed more spread tubulin fibers (Figure 5B). Conversely, transfection of the FERM domain induced increased tubulin fiber assembly (Figure 5A), accompanied by longer (Figure 5E), thicker (Figure 5D), and more aligned tubulin fibers (Figure 5F), which displayed a more spread structure (Figure 5B). Cells transfected with inactive ezrin T567A shared almost the same structural properties with cells expressing wild-type ezrin.

**Figure 6:**
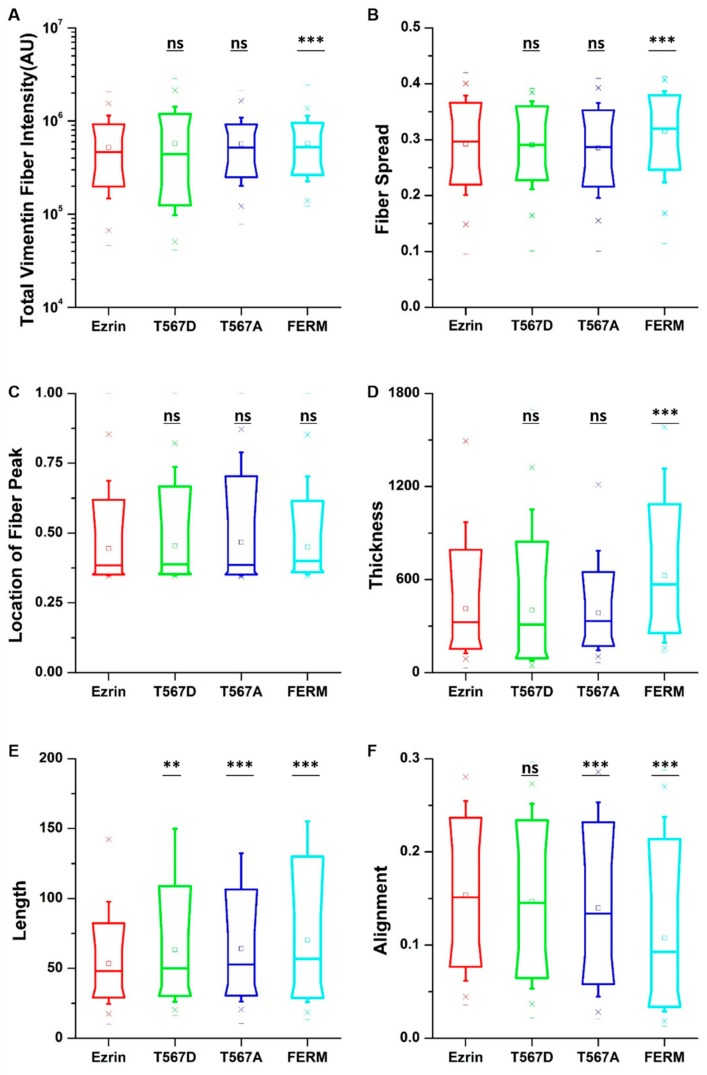
The effect of ezrin mutations on the vimentin cytoskeleton. Box plots show the results of the total vimentin fiber intensity (**A**), fiber spread (**B**), location of the fiber peak (**C**), fiber thickness (**D**), fiber length (**E**), and fiber alignment (**F**). Box plots extend from the 10th to the 90th percentile, whiskers from the 5th to the 95th. A total of n = 585 (ezrin), n = 424 (ezrin T567D), n = 768 (ezrin T567A), and n = 425 (FERM) cells were analyzed from n = 6 repeats. Asterisks indicate a statistical difference (** *p* < 0.01, *** *p* < 0.001, obtained using Dunnett’s test against wild-type ezrin).

When assessing the vimentin structure (Figure S7), we found that active ezrin T567D increased the vimentin fiber length (Figure 6E). Also, the dominant negative FERM domain induced longer (Figure 6E), thicker (Figure 6D), more aligned (Figure 6F), and more spread vimentin fibers (Figure 6B). Finally, inactive ezrin T567A had little effect on vimentin organization. Together, our results show that ezrin mutations have a collective and pronounced effect on actin, tubulin, and vimentin structure, with effects that appear in parallel for all three structures.

### 2.5. The Effect of Ezrin Mutations on Cellular Mechanical Properties

**Figure 7:**
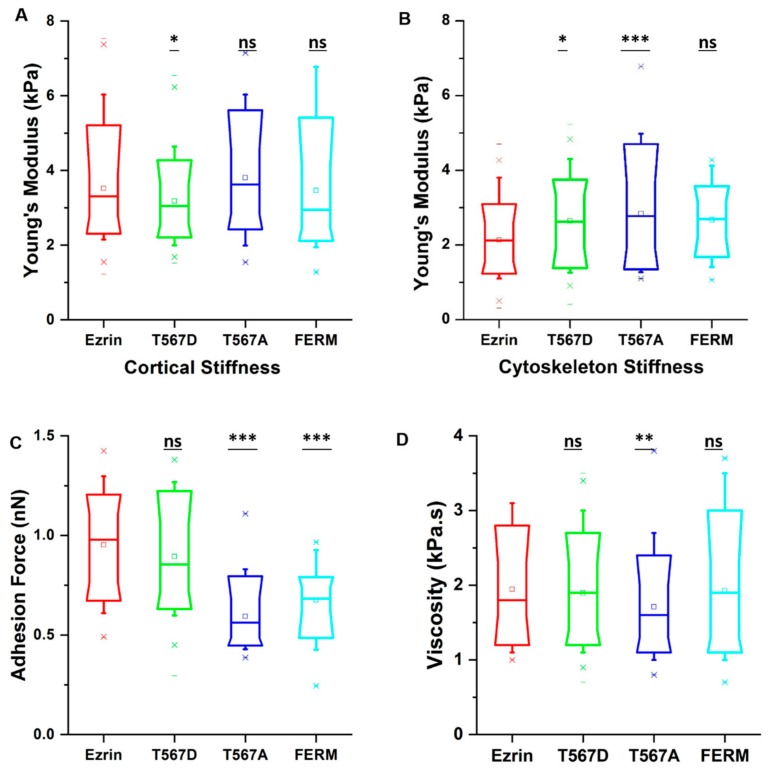
The effect of ezrin mutations on the cell mechanics. Box plots show the results of the migration cortical stiffness (**A**), cytoskeleton stiffness (**B**), adhesion force (**C**), and viscosity (**D**). Box plots extend from the 10th to the 90th percentile, whiskers from the 5th to the 95th. A total of n = 125 (ezrin), n = 115 (ezrin T567D), n = 89 (ezrin T567A), and n = 74 (FERM) cells were analyzed from n = 6 repeats. Asterisks indicate a statistical difference (* *p* < 0.05, ** *p* < 0.01, *** *p* < 0.001, obtained using Dunnett’s test against wild-type ezrin).

Finally, having established the effects of different ezrin mutants on cell migration and cytoskeletal organization, we employed atomic force microscopy (AFM) to verify whether the observed changes also lead to changes in the mechanical properties. Force indentation curves were taken by probing only transfected cells, as selected from the epifluorescence camera. Given that ezrin’s primary role is as a linker between the membrane and the cortical actin network, we chose to separately analyze the stiffness associated with the cell cortex and that of the stress fiber-rich underlying cytoskeleton. In addition, we were able to obtain the value of the adhesion force and viscosity. Interestingly, we found that expression of active ezrin T567D led to a decrease in cortical stiffness (Figure 7A) while both active ezrin T567D and inactive ezrin T567A increased cytoskeleton stiffness (Figure 7B) compared with wild-type ezrin. The dominant negative FERM domain had no effect on cell stiffness (Figure 7A,B). In line with results by others [2], cells transiently transfected with inactive ezrin T567A and dominant negative FERM domain had a significantly lower adhesion force (Figure 7C), while active Ezrin had little effect on this property. Surprisingly, cells expressing inactive ezrin T567A showed decreased viscosity compared with wild-type ezrin (Figure 7D).

## 3. Discussion

In this study, we investigated the role of ezrin, a linker between the cell membrane and actin cytoskeleton, in the relationship between cell migration and cells’ biophysical properties. Our results show that cells transfected with active ezrin T567D display an enhanced migration speed and directionality, decreased cortical stiffness, and increased cytoskeleton stiffness (Supplementary Table S1).

Polarization of the intracellular localization of some key proteins is thought to be a prerequisite for cell migration, which may then determine the direction of movement. Interestingly, we found that active ezrin T567D displayed highly polarized intracellular localization, with more protein accumulating at the cell rear. In this connection, both ezrin and phosphorylated moesin, another member of the ERM, provide a ‘directional memory’ to migrating cells by preferentially accumulating at the cell rear in chemotaxis experiments, and remaining polarized even after the removal of the chemoatractant. We find a similar behavior, specifically for active ezrin, whose overexpression enhances the directionality of migrating cells. In our images, the rear structure often looks like a uropod, as typically observed in amoeboid migration (Figure 2A). Therefore, and unlike previous studies [3,30], the strengthening of the cell–cortex connection is highly localized only at the cell rear while the remaining cell periphery may be mechanically weaker and more likely to display protrusions. This hypothesis would then explain why we observed a significantly softer cell cortex and lower but not significant adhesion forces for T567D transfections, given that our measurements averaged the mechanical properties of the whole cell cortex. Additionally, we found that the more active ezrin accumulates at the cell rear, the faster cells migrate. Therefore, optimal directed migration would involve an opposing modulation of mechanical properties at the rear and front of the cell, requiring not only activation of ezrin but also its finely controlled accumulation at the rear and depletion anywhere else at the cell membrane. It remains unclear whether the activation of ezrin takes place preferentially at the rear or the activation process leads to a rear translocation of ezrin. In this connection, previous studies have reported that ezrin T567D has a higher affinity for membrane-bound PIP2 [31] and is less mobile within the membrane [32] than its wild-type counterpart. Accordingly, once the preferential localization of active ezrin is initially established at what becomes the cell rear, ezrin localization likely remains polarized, thus giving rise also to a stably soft cortex with frequent membrane protrusions everywhere but at the rear. Of note, this study was carried out in fibroblasts, which due to their mesenchymal nature, become well spread and display a strong migratory phenotype when cultured in sparse conditions. This makes them a suitable model to study the relationship between cell migration direction and cytoskeletal organization or intracellular distribution of proteins. Additional studies on, e.g., epithelial cells should provide a further understanding on the role of ezrin’s phosphorylation in cell monolayer models, where cell–cell adhesions play a key role or where epithelial-to-mesenchymal transitions may be studied.

A number of studies have focused on characterizing the relationship between cell stiffness and cell motility. In particular, higher cell motility is typically associated with lower stiffness in both cancer cells [33] and non-cancer cells [34]. Conversely, we used depth-dependent approaches to analyze our AFM force–indentation curves, estimating both the stiffness of the cortex and that of the underlying cytoskeleton [24]. In our experiments, cells with higher motility had a softer cell cortex, which may benefit migration due to its increased tendency to extend protrusions, which then become the leading edge of the migrating cell. Simultaneously, the underlying cytoskeleton displayed higher stiffness, which may illustrate an unreported feature leading to increased migratory capacity. In this connection, another requirement of optimal migration is to efficiently push the nucleus forward, or deform it through pores in the particular situation of 3D migration. Accordingly, our results for T567D suggest a well-anchored nucleus that is pulled (increased volume) by the reinforced cytoskeleton (increased cytoskeletal Young’s modulus). Of note, and even though it is not reflected in our 2D migration experiments, the increased auxetic properties of the nucleus for T567D transfection would allow the cell to better steer it’s nucleus through the pores of the extracellular matrix, given that the compressive forces imposed by the extracellular environment would lead to an overall decrease in the nuclear volume.

Transfection with inactive ezrin T567A, which binds to the membrane but cannot bind to actin, induces opposite effects to T567D, rather than just replicating the wild-type phenotype. For example, others have shown that T567D impairs cell migration and weakens the connection between the membrane and the actin cortex [2,29,35]. We observed similar results, even though the decrease in migration speed was not significant in our experiments. Furthermore, we also report increased stiffness, alongside decreased viscosity and the accumulation of actin fibers, which likely reflects a shift away from the fluid-like mechanical phenotype typically associated with the metastatic potential of malignant cells [36]. Interestingly, our results also show that inactive ezrin T567A accumulates around the cell nucleus, forming a ring, and that the nucleus is subjected to reduced intracellular pulling as well as being less auxetic. In this connection, it is reported that the lack of nucleus deformability limits the migration speed through 3D tissues [37]. From a clinical perspective, cervical cancer patients displaying perinuclear ezrin localization patterns have longer survival times than those with a more diffuse cytoplasmic ezrin localization [38]. Put together, we hypothesize that the inactivation of ezrin is linked to a transition towards a solid-like behavior of the cytoskeleton and a reduced ability of the cell to move its nucleus through a crowded extracellular environment, which, in the case of cancer, would hinder malignant cells from migrating through their host tissue.

The most life-threatening events in cancer progression are invasion and metastasis [39], which cause the vast majority of cancer deaths. Here, we found that overexpression of phosphorylated ezrin resulted in increased cell migration. This is consistent with previous studies showing that the invasive and metastatic ability of cancer cells is linked to high expression of phosphorylated ezrin [20,21]. We propose that overexpression of phosphorylated ezrin is associated with a reorganization of the cellular cytoskeleton, which leads to a decrease in the cortical stiffness and an increase in the cytoskeleton stiffness. Collectively, these biophysical changes then support a more migratory phenotype. Together, our results highlight the importance of phosphorylated ezrin as a biomarker for cancer metastasis diagnosis. Similarly, and even though further studies are needed to understand the molecular mechanism in detail, our results suggest that ezrin phosphorylation might be a promising molecular target for cancer therapy, especially to suppress cancer invasion and metastasis.

## 4. Materials and Methods

### 4.1. Cell Culture and Transfection

Ezrin is composed of three distinct domains: An N-terminal cell membrane-binding domain, which is also called the FERM domain; the central α-helical domain; and the C-terminal actin filament-binding domain. In its inactive mode, the FERM–C terminal interaction masks both the cell membrane and actin filament-binding sites, resulting in ezrin’s cytosolic localization. The activation of ezrin has been proposed to follow a two-step mechanism, involving first FERM domain binding to phosphatidylinositol-4, 5-bisphosphate (PIP2) at the membrane, followed by the phosphorylation of threonine 567 by Rho-kinase [40] or PKC isoforms [34]. To explore the role of ezrin T567 phosphorylation, we used different plasmids that reflect the different activation states of ezrin. In particular, we used plasmids of wild-type ezrin and its mutants ezrin T567A (constitutively inactive actin binding), ezrin T567D (constitutively active actin binding and impaired head to tail association), and FERM domain (N-terminal domain of ezrin), which were a kind gift from Prof. Guillaume Charras (University College London, London, UK).

NIH3T3 cells were used as cell model and cultured in Dulbecco’ modified eagle medium (DMEM, Thermo Fisher, Waltham, MA, USA) supplemented with 10% fetal bovine serum (FBS, Sigma, St. Louis, MO, USA). Cells were grown in a humidified incubator with 5% CO_2_ at 37 °C. In all experiments, cells were seeded the day before transfection to reach 80% to 90% confluence at the time of transfection. All transfections were carried out with Lipofectamine LTX Plus (Thermo Fisher, Waltham, MA, USA). For 1 × 10^5^ cells, 250 ng of plasmid were used. After 6 h, transfection medium was replaced by normal growth medium. The transfection efficiency was approximately 60%. All the experiments were performed on visibly transfected cells only, by confirming their fluorescence intensity levels through optical imaging (Figure S1).

### 4.2. Immunocytochemistry

Transfected cells were seeded at low density one day prior to fixation. Cells were fixed with 3.7% paraformaldehyde (Sigma) for 10 min, permeabilized with 0.25% Triton X-100 (Sigma) for 5 min, and blocked with 10% BSA (Sigma) for 1 h at room temperature. Samples were incubated overnight at 4 °C with primary antibodies against tubulin (1:200, Abcam ab4074), vimentin (1:200, SantaCruz sc373717), or ezrin (1:200, SantaCruz sc58758). Secondary antibodies anti-Rabbit (1:500, Abcam ab6718), anti-rabbit (1:500, Invitrogen a21206), anti-mouse (1:500, Invitrogen a21202), and anti-mouse (1:500, Invitrogen a31570) were subsequently incubated for 1 h at room temperature. Tetramethylrhodamine (TRITC) or fluorescein isothiocyanate (FITC) coupled phalloidin (1:500, SantaCruz sc301530, sc363791) were used to stain the actin filaments. After staining, coverslips were mounted onto glass slides using ProLong Gold Antifade Mounting medium containing DAPI (Thermo Fisher, Waltham, MA, USA). Images of the fixed samples were obtained using an inverted epifluorescence microscope (Leica DMI4000B, Wetzlar, Germany) with a × 20/0.5 NA objective lens and a CCD camera (Leica DFC300FX, Wetzlar, Germany). Single cells that were not damaged or mitotic were chosen for imaging. Cells were sequentially imaged on TRITC, FITC, and DAPI channels for later analysis.

### 4.3. Image Quantification Analysis

The pipeline for single cell quantification of the cytoskeleton and nuclear structures has been described in detail elsewhere [26,30,41]. Briefly, the algorithm uses grey-scale immunostaining-based images and it follows three independent steps: (1) Initial fiber segmentation, (2) fiber refinement, and (3) determination and subtraction of the non-uniform background within the cell boundaries. More details on these steps can be found in the associated Supplementary Material. The algorithm outputs data at the single cell level, including gross cell morphology information, like the cell area, aspect ratio, and stellate factor; cytoskeleton information, like the fiber intensity, length, and thickness; and nucleus data, such the as relative volume and Poisson’s ratio. A very detailed description of these parameters can be found in a previous publication from our group [27].

### 4.4. Cell Migration Time-Lapse Experiments

Transfected cells were seeded onto a 6-well plate at low density. Prior to imaging, the medium was replaced with FBS-supplemented Flurobrite-DMEM imaging-specific medium (Thermo Fisher, Waltham, MA, USA) to reduce background fluorescence and photobleaching. Time-lapse recordings of single cell dynamics were acquired with a 20× objective by a Lumascope LS720 microscope (Etaluma, Carlsbad, CA, USA) at a rate of 1 image every 5 min for at least 12 h. The miniaturized microscope was placed inside the incubator, so the temperature and CO_2_ concentration were maintained throughout the time-lapse experiment.

The algorithm to analyze time-lapse fluorescence videos is based on grey-scale images of the fluorescent channels and there are three steps: (1) Determination of the cell outlines for every frame; (2) calculation of the positions of cell centroids, instantaneous velocity, and direction; and (3) quantification of the protein distribution parameters, i.e., the polarization ratio and peak front-to-back ratio with respect to the instantaneous direction of migration. Once the position of the cell’s centroid was determined for each frame, we computed the cell’s instantaneous migration speed and the directionality of the overall recorded migration path as previously described elsewhere [27].

### 4.5. AFM Measurements of Mechanical Properties

Cells transfected with one of the plasmids were plated on petri dishes in FBS and HEPES-supplemented medium (Thermo Fisher, Waltham, MA, USA). All experiments were conducted on a Nanowizard 4 AFM (JPK, Berlin, Germany) with a petri dish heater to maintain the samples at 37 °C. Rectangular gold-coated silicon nitride cantilevers with four-sided pyramidal tips (0.06 N/m spring constant, Budget Sensors) were used as probes. Before each experiment, the spring constant of the cantilever was calibrated using the thermal fluctuations method. Briefly, the cantilever sensitivity was calculated based on a force curve on a bare region of the petri dish, followed by calibrating the force constant by thermal fluctuations. Fluorescent images of the transfected cells were acquired using an Axio Observer Z.1 epifluorescence microscope with a Plan-Apochromat lense (20×) and a water-cooled CMOS camera (Orca Flash 4, Hamamatsu Photonics, Hamamatsu City, Japan). Mechanical measurements were performed using JPK’s QI mode, which rapidly acquires force curves, generating a detailed image of the topography and mechanical properties of the sample. Data analysis of the force–displacement curves was carried out using the Bottom Effect Cone Correction (BECC) model for thin adherent cells on a stiff substrate using a pipeline written in MATLAB as previously described [41,42].

The algorithm used to calculate the stiffness of the cell cortex and the deeper cytoskeleton from a single force curve was proposed by Lekka’s group and is described elsewhere [43]. Similarly, the algorithm to compute the cellular viscosity from single indentation curves was proposed by Radmacher’s group and can be found elsewhere [44].

### 4.6. Statistical Analysis

Statistical analysis was performed with OriginLab analysis software. Results were statistically analyzed by one-way analysis of variance followed by Dunnett test against the control (wild-type ezrin transfection). Statistical significance was reported as *p* < 0.05 (*), *p* < 0.01 (**), and *p* < 0.001 (***) unless otherwise stated.

## Supplementary Materials

Supplementary materials can be found at https://www.mdpi.com/1422-0067/21/2/435/s1.

## Abbreviations

ERM	Ezrin/radixin/moesin
AFM	Atomic force microscopy
