# Supplementary material for: Ezrin Phosphorylation at T567 Modulates Cell Migration, Mechanical Properties, and Cytoskeletal Organization
Source: Int J Mol Sci. 2020 Jan 9;21(2):435. doi: 10.3390/ijms21020435 (PMC7013973; doi:10.3390/ijms21020435)
Supplement: Supplementary file 1 [file ijms-21-00435-s001.pdf]

International Journal of
Molecular Sciences

MDPI

Supplementary information

Ezrin phosphorylation at T567 modulates cell migration, mechanical properties and cytoskeletal organization

Xiaoli Zhang[1], Luis R. Flores[1], Michael C. Keeling[1], Kristina Sliogeryte[1], Núria Gavara[1]*

[1] School of Engineering and Material Science, Queen Mary University of London, Mile End Road, E1 4NS, London, UK

* Correspondence: n.gavara@qmul.ac.uk; Tel.: +44(0) 207 882 6596

Supplementary Table 1. Summary of main changes due to different Ezrin mutations in the biophysical properties measured in this study. (+) indicates significant increase, (-) indicates significant decrease and (=) indicates no significant changes.

**Biophysical parameter**	**T567D**	**T567A**	**FERM**
Migration velocity	+	=	=
Migration directionality	+	=	=
Rear localization	+	+	=
Cortical stiffness	-	=	=
Cytoskeletal stiffness	+	+	=
Viscosity	=	-	=
Adhesion strength	=	-	-
Cell area	-	=	-
Membrane projections/lamellipodia	+	+	=
Nuclear volume	+	-	-
Nuclear auxetic properties	+	-	-
Stress fibre assembly	=	+	=
Microtubules assembly	=	=	+
Vimentin assembly	=	=	+

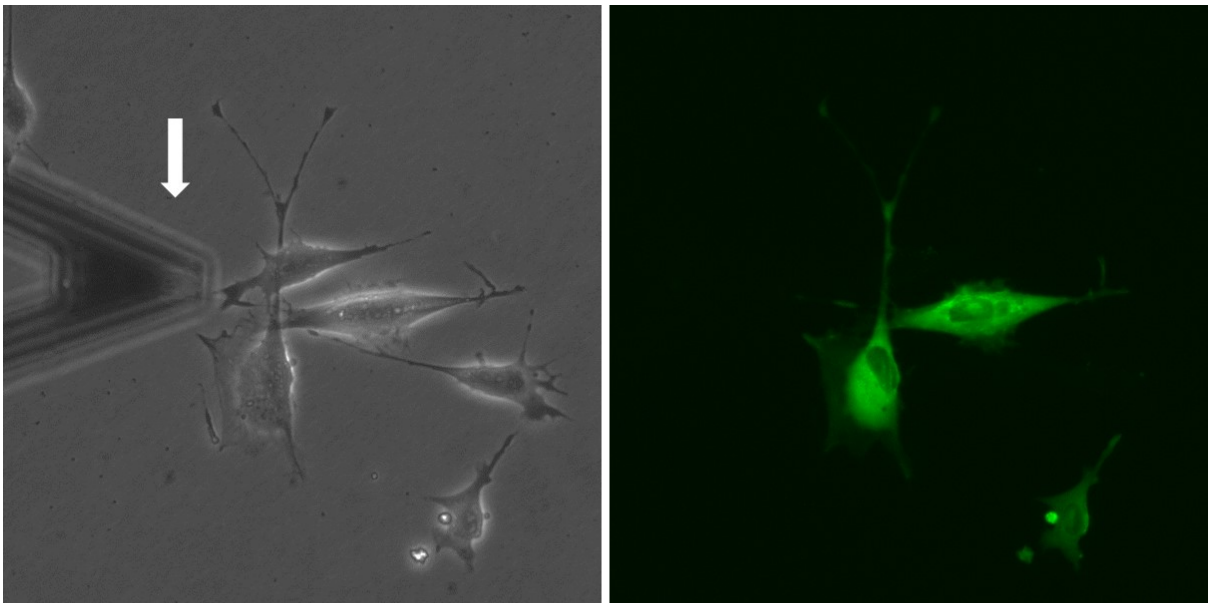

Supplementary Figure S1. Example images for AFM measurement. Left: bright field images of cells, arrow indicates the cantilever; right: fluorescent image of cells transfected with inactive Ezrin T567A. Scale bar μm.

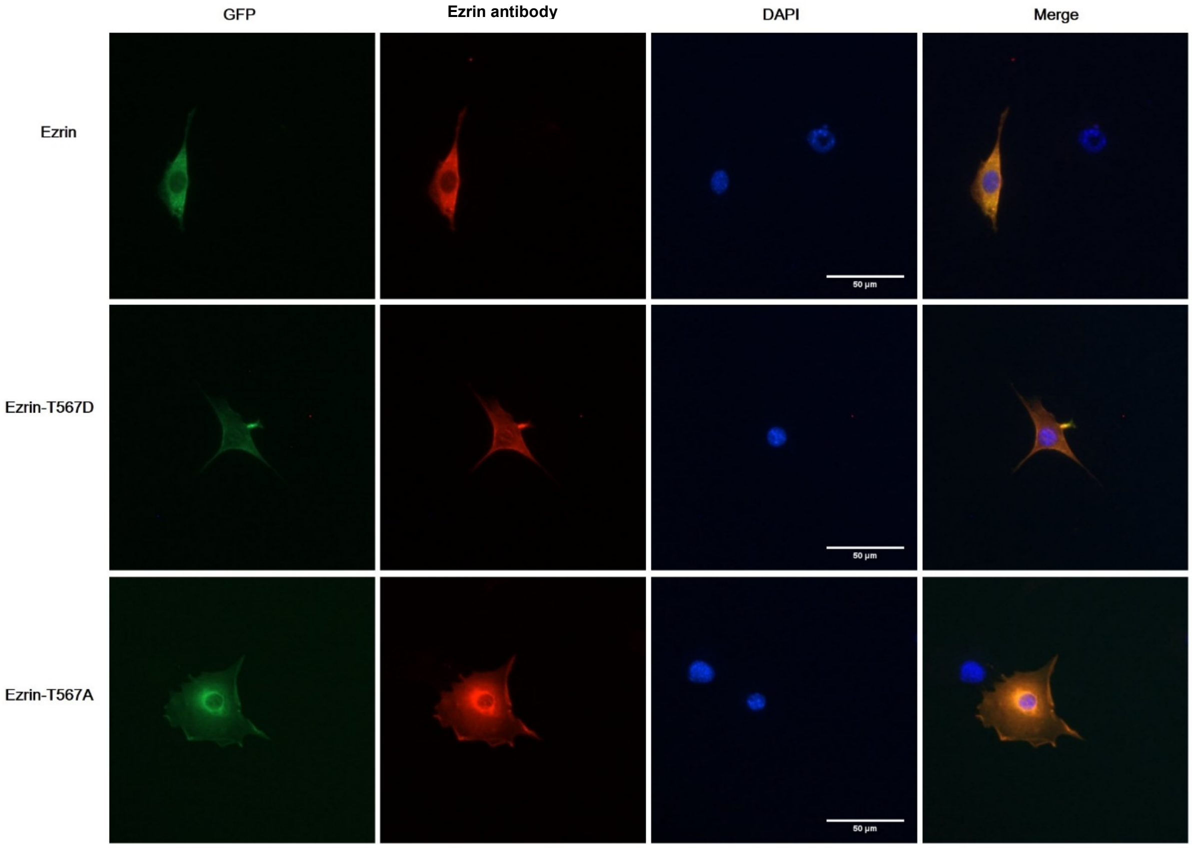

Supplementary Figure S2. Fluorescent images of transfected cells co-stained with Ezrin antibody and DAPI. Left: transfected cells expressing Ezrin mutant coupled with GFP; 2nd column: Ezrin stained with Ezrin antibody; 3rd column: cell nuclear stained with DAPI; right: merged images for the three channels. Scale bar 50 μm.

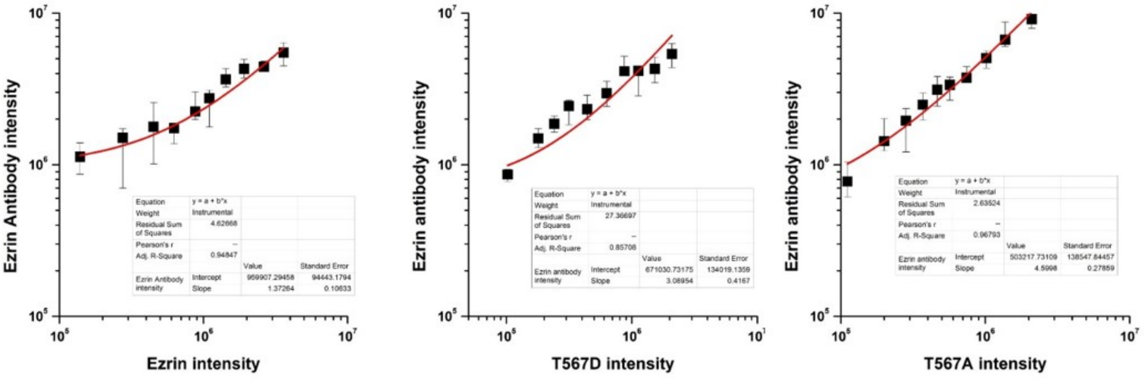

Supplementary Figure S3. Levels of Ezrin transfection. Plots of total fluorescence intensity obtained through transfected GFP- or RFP-Ezrin versus total fluorescence intensity obtained through immunostaining using an antibody against Ezrin. The fits are used to measure the ratio between endogenous and exogenous Ezrin amount in our transfections.

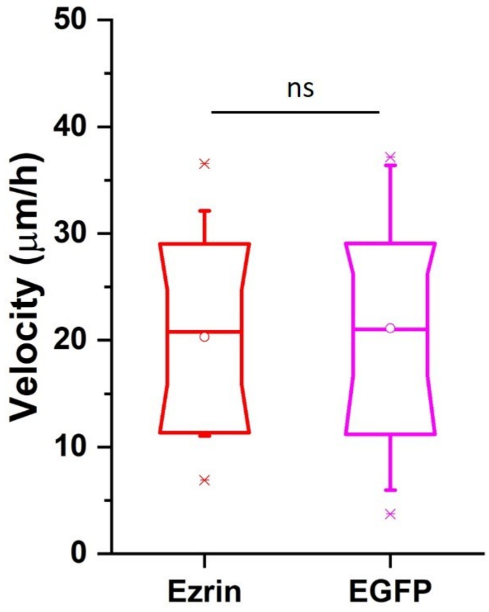

Supplementary Figure S4. Transfection of Ezrin had no effect on cell migration compared with cells transfected with EGFP. Box plot show the results of migration velocity, n=21 (Ezrin), n=34 (EGFP). Box plot extends from the 10th to the 90th percentile, whiskers from the 5th to the 95th. Ns indicate no significant difference, measured using a t-test.

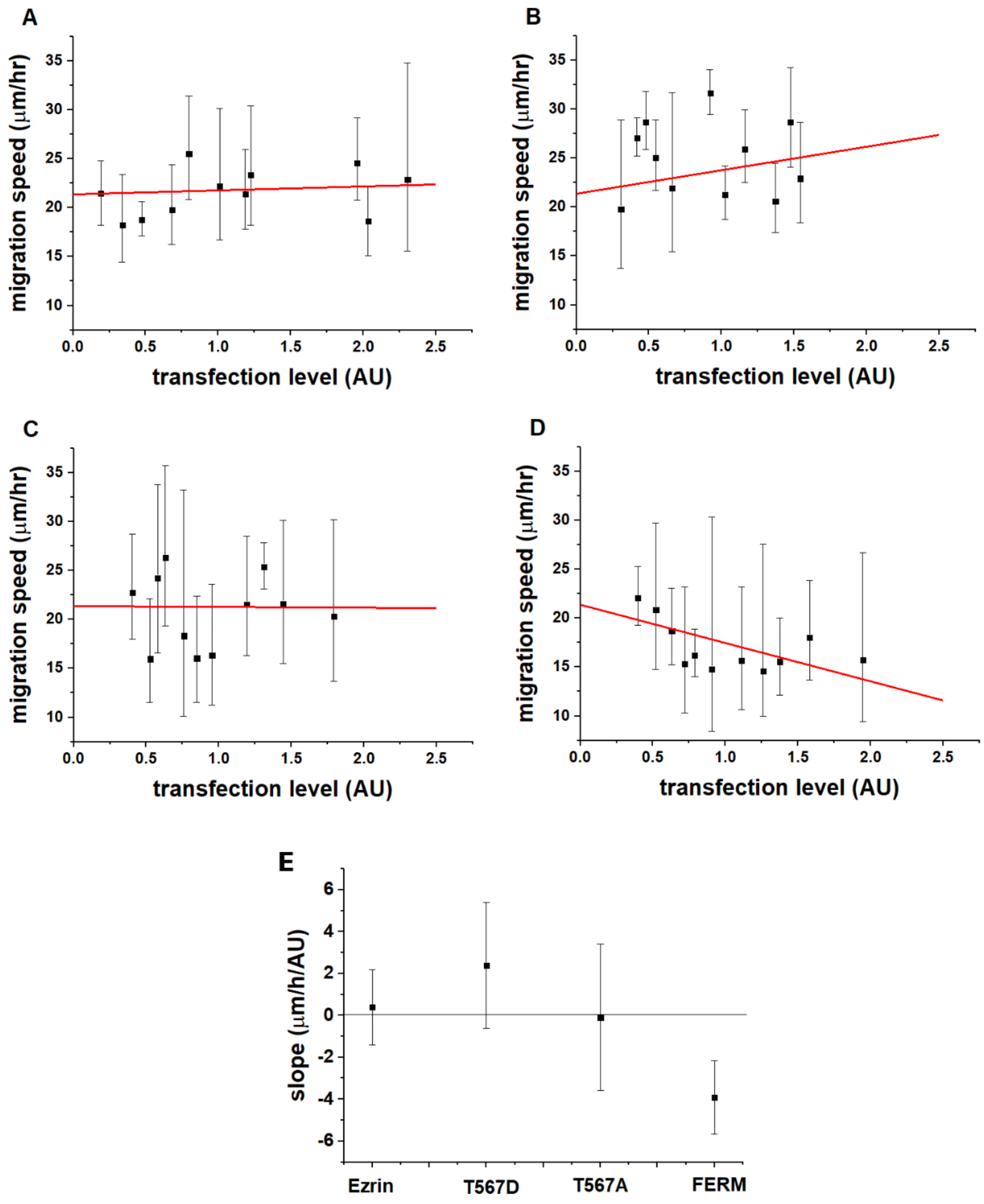

Supplementary Figure S5. The amount of exogenous protein has a weak effect on the observed changes in cell migration. Scatter plots show the results of migration velocity for cells pooled and averaged according to their exogenous protein expression levels, for transfections with Ezrin (A), T567D (B), T567A (C) and FERM (D). Error bars extends to 25th and 75th percentile, and fit lines correspond to a linear fit. Panel E shows the magnitude and SD for the slopes of the fitted lines as obtained in panels A-D.

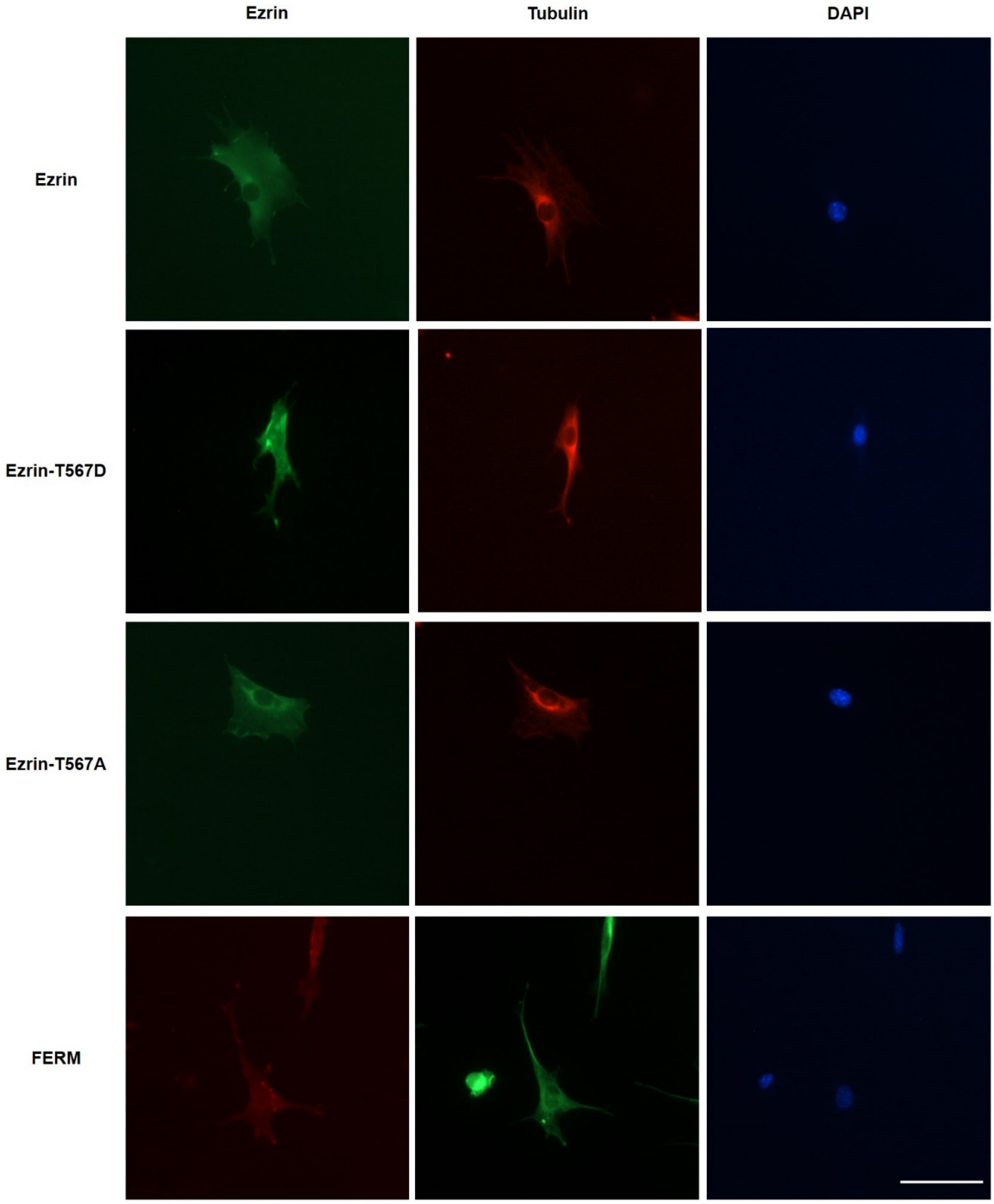

Supplementary Figure S6. Fluorescent images of transfected cells co-stained with Tubulin antibody and DAPI. Left: transfected cells expressing Ezrin mutant coupled with GFP or RFP; middle column: microtubules stained with tubulin primary antibody and TRITC (for Ezrin WT, T567D and T567A) or FITC (for FERM) secondary antibody; right column: cell nuclear stained with DAPI. Scale bar 50 μm.

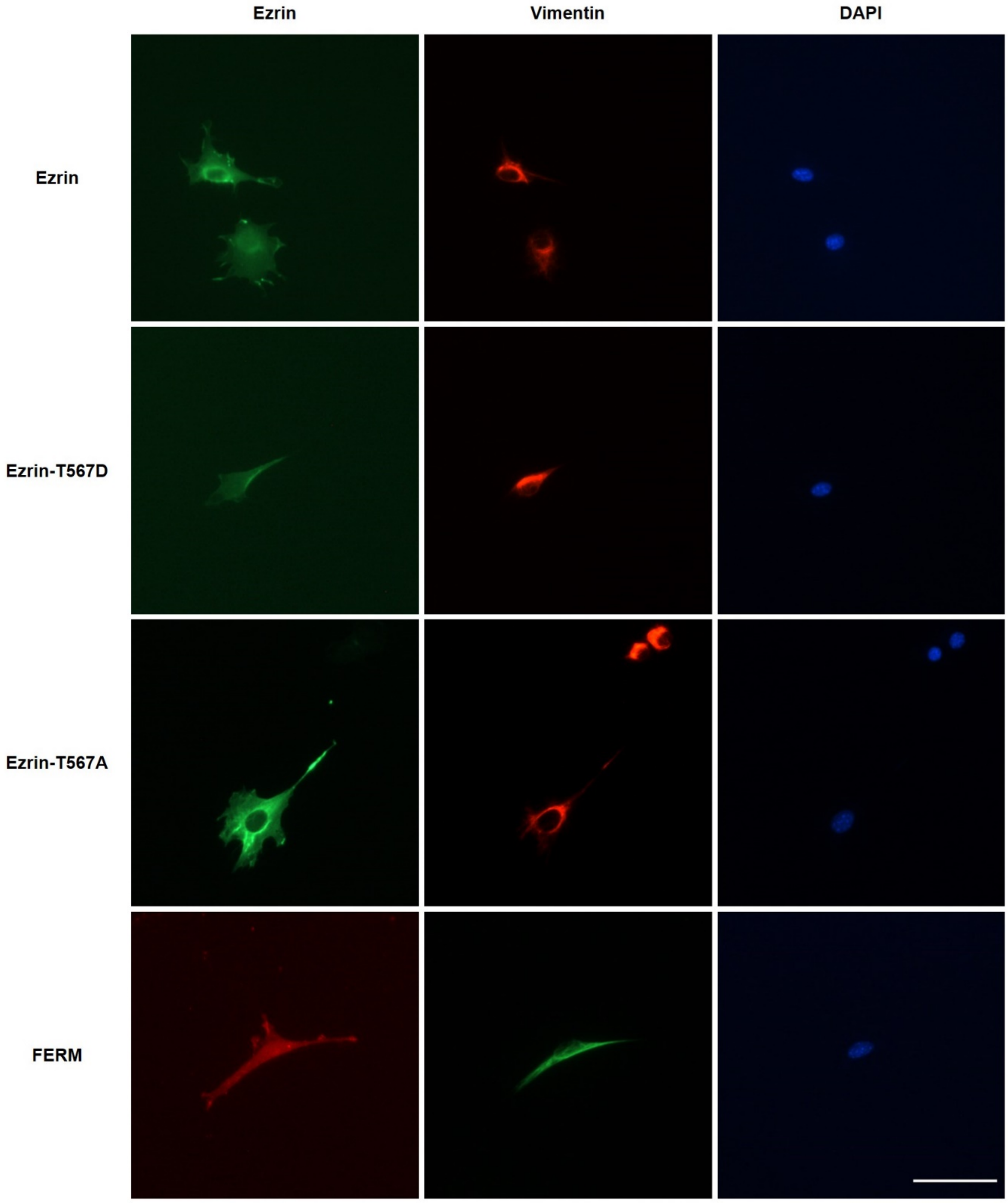

Supplementary Figure S7. Fluorescent images of transfected cells co-stained with vimentin antibody and DAPI. Left: transfected cells expressing Ezrin mutant coupled with GFP or RFP; middle column: vimentin fiber stained with vimentin primary antibody and TRITC (for Ezrin WT, T567D and T567A) or FITC (for FERM) secondary antibody;; right column: cell nuclear stained with DAPI. Scale bar 50 μm.

Procedure for single cell analysis of immunofluorescence images

The pipeline uses grey-scale immunostaining-based images and it follows five independent steps:

(1) Determination of the cell outlines for every single cells: Because images were taken with 20x objective and cell sizes are small so there are normally many cells in one single frame. The first step is that single cells, which are not attached with other cells, are selected and cropped by a rectangular area. Figure S8 A-C show examples of cropped images with different channels. The outline of the cell is identified by detecting the difference between signals inside and outside the cells. In order to obtain accurate cell boundary, an outline around the cell perimeter is drawn manually on the image. Then a cell mask is automatically generated by using the algorithm (Figure S8D);

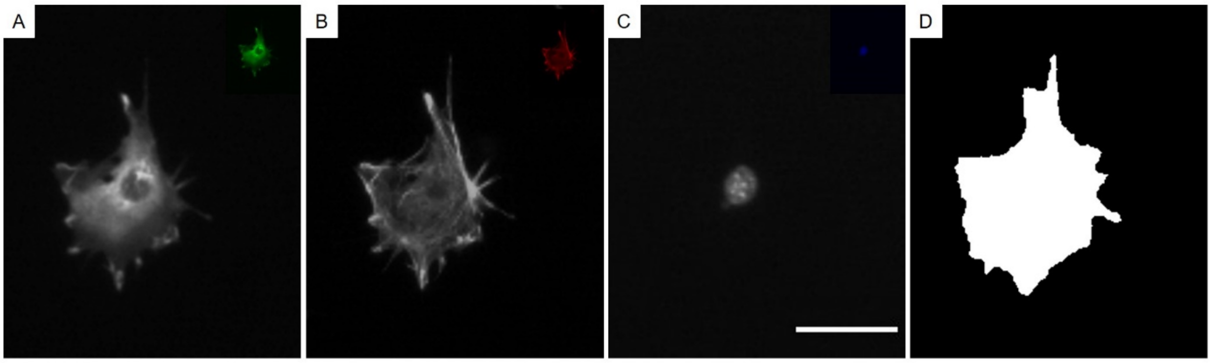

Supplementary Figure 8: Example inputs and outputs of the image analysis pipeline. Images of cells transfected with Ezrin (A) stained with actin filaments (B) and DAPI (C) are used as inputs to calculate Ezrin properties, cytoskeleton morphometric parameters and nuclear properties, respectively; D. Outline of the cell is generated from these images to identify the perimeter of a single cell. Scale bar: 30 μm

(2) Initial fiber segmentation: After identification of cell outline, cytoskeleton fibers are determined by convolution of the cell image with elongated Laplace of Gaussian (eLoG) kernels [1]. A small window with a size of 21x21 pixel working as a fiber template filter is applied at each pixel location within the cell. The window is rotated 180° with a step size of 6° gradually and a map of putative fibers is generated by the maximization of image cross correlation signals

(3) Fiber refinement: Fiber refinement is then carried out using a coherence enhancing diffusion filter. The fiber map identified in step two is refined by extension and interconnection of interrupted fibers. Then the values of newly inserted pixels are examined by comparing with the average orientation of neighboring pixels within the same fiber, using a 9 x 9 pixel window. The pixel is discarded if the difference is larger than pre-defined threshold. Also this step would correct artifacts like bright dots. These fiber-enhancement and trimming steps are iterated until convergence of the algorithm.

(4) Subtraction of background: At last, fluorescent signals of non-specific binding that do not belong to the fibers are determined and removed by the pipeline. A background fluorescence map is generated by computing median signal intensity within a 21 x 21 window surrounding each non-fiber pixel near fiber edges. The result is a smoothly changing intensity map with fiber pixels replaced by the median of non-fiber pixels. This background map is then subtracted from the original image and the pixels that obtained negative values belonging to a fiber map are removed and the process is iterated until convergence is reached. This process ensured that only pixels truly belonging to a fiber are included in the resulting fiber map.

(5) Parameter calculations: 18 parameters are calculated characterizing the cytoskeleton and nuclear properties. Figure S9 shows an example of analyzed actin fibers using this pipeline method and outputs of the actin fiber maps

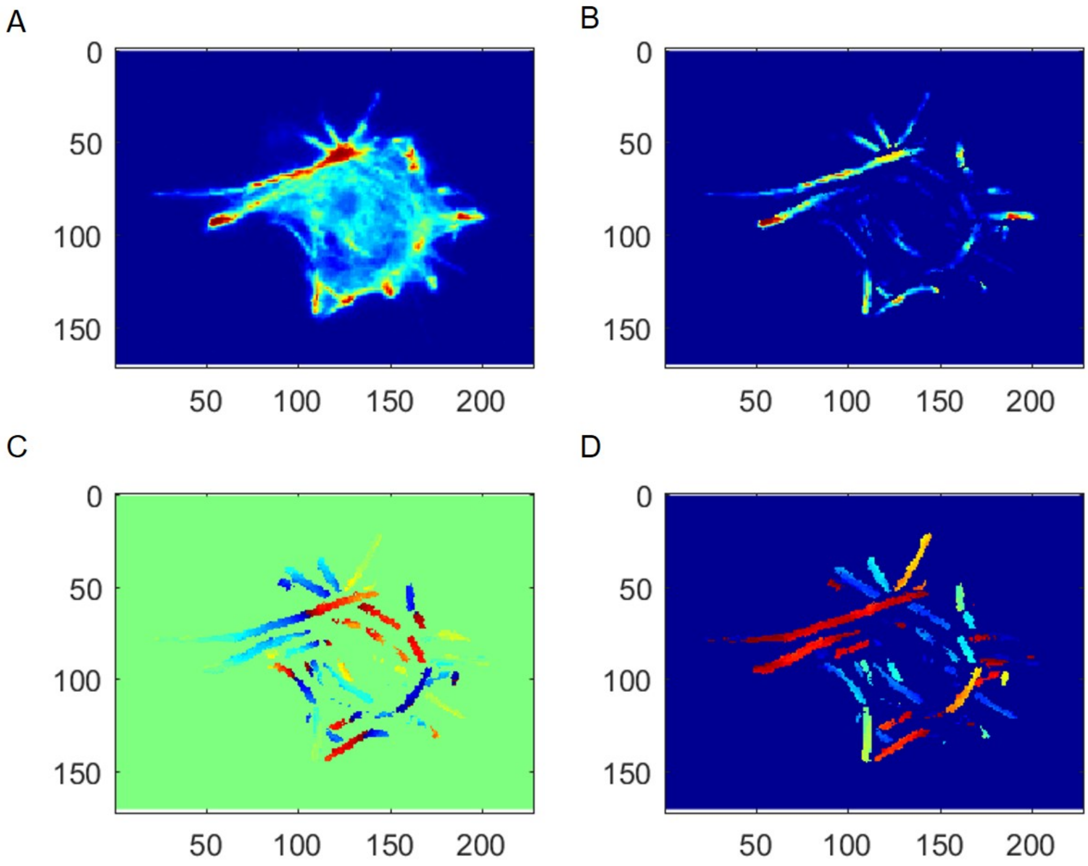

Supplementary Figure 9: Example outputs of the actin fiber maps using the pipeline method. A and B represent the hot map and actin fibers identified by the pipeline; while C and D represent map angular orientation of actin fibers. More detailed information can be found in references [2-4].

[1] A. Zemel, F. Rehfeldt, A. Brown, D. Discher, S. Safran, Optimal matrix rigidity for stress-fibre polarization in stem cells, Nature physics 6(6) (2010) 468.
[2] N. Gavara, R.S. Chadwick, Relationship between cell stiffness and stress fiber amount, assessed by simultaneous atomic force microscopy and live-cell fluorescence imaging, Biomechanics and modeling in mechanobiology 15(3) (2016) 511-523.
[3] M.C. Keeling, L.R. Flores, A.H. Dodhy, E.R. Murray, N. Gavara, Actomyosin and vimentin cytoskeletal networks regulate nuclear shape, mechanics and chromatin organization, Scientific Reports 7(1) (2017) 5219.
[4] L.R. Flores, M.C. Keeling, X. Zhang, K. Sliogeryte, N. Gavara, Lifeact-GFP alters F-actin organization, cellular morphology and biophysical behaviour, Scientific reports 9(1) (2019) 3241.
